# A Comparative Analysis of the Mechanism of Toll-Like Receptor-Disruption by TIR-Containing Protein C from Uropathogenic *Escherichia coli*

**DOI:** 10.3390/pathogens5010025

**Published:** 2016-02-29

**Authors:** Anna Waldhuber, Greg A. Snyder, Franziska Römmler, Christine Cirl, Tina Müller, Tsan Sam Xiao, Catharina Svanborg, Thomas Miethke

**Affiliations:** 1 Institut für Medizinische Mikrobiologie, Immunologie und Hygiene, Technische Universität München, Trogerstr. 30, München 81675, Germany; Anna.Waldhuber@med.uni-muenchen.de (A.W.); franziska.roemmler@tum.de (F.R.); ccirl@gmx.de (C.C.); tina.mueller@tum.de (T.M.); 2 Institute of Human Virology, Departments of Medicine, and Microbiology and Immunology, University of Maryland, School of Medicine, 725 West Lombard Street, Baltimore, MD 21201, USA; GSnyder@ihv.umaryland.edu; 3 Department of Pathology, Case Western Reserve University, 2103 Cornell Rd, Cleveland, OH 44106, USA; tsan.xiao@case.edu; 4 Department of Microbiology, Immunology and Glycobiology (MIG), Institute of Laboratory Medicine, Lund University, 221 00 Lund, Sweden; catharina.svanborg@med.lu.se; 5 Institute of Medical Microbiology and Hygiene, University of Heidelberg, Theodor-Kutzer-Ufer 1-3, Mannheim 68167, Germany

**Keywords:** bacterial pathogens, Toll-like receptors, TIR-containing proteins, TIR-domain structure

## Abstract

The TIR-containing protein C (TcpC) of uropathogenic *Escherichia coli* strains is a powerful virulence factor by impairing the signaling cascade of Toll-like receptors (TLRs). Several other bacterial pathogens like *Salmonella*, *Yersinia*, *Staphylococcus aureus* but also non-pathogens express similar proteins. We discuss here the pathogenic potential of TcpC and its interaction with TLRs and TLR-adapter proteins on the molecular level and compare its activity with the activity of other bacterial TIR-containing proteins. Finally, we analyze and compare the structure of bacterial TIR-domains with the TIR-domains of TLRs and TLR-adapters.

## 1. Introduction

Uropathogenic *Escherichia coli* (UPEC) strains are the leading cause of urinary tract infections. The frequent disease affects almost half of all women during their lifetime and is increasingly difficult to treat since resistance against any class of antibiotics spreads globally with alarming speed [1,2,3]. To counter this emerging thread, the exploration of host–pathogen interactions may reveal potential new drug targets to combat infections. The initial step of a host–pathogen interaction resides in the recognition of pathogens by innate immune cells initiating defense programs of the host finally leading to the elimination of the invading pathogen. Toll-like receptor (TLR) 4 is an important recognition element for UPECs by controlling immune responses such as chemo- and cytokine release and recruitment of polymorphonuclear granulocytes. To reduce or avoid immune responses UPECs secrete a virulence factor, which we designated TIR-containing protein C (TcpC). TcpC is capable to transverse cell membranes and interferes with the detection of the microorganism by TLRs. It contains a Toll/Interleukin-1 receptor (TIR) domain, which is characteristic for TLRs and their adaptor molecules as well as the Interleukin-1 receptor. TcpC binds to myeloid differentiation factor 88 (MyD88), the central adaptor of the TLR signaling cascade, but also to TLR4. In this review, we summarize the mechanism of TLR impairment by TcpC in detail and compare TcpC with TIR-containing proteins (Tcps) from other bacteria.

## 2. Results and Discussion

### 2.1. The TIR-Containing Protein C and Its Relatives

TLRs comprise a family of 10 functional proteins in humans, 12 in mice, and recognize pathogen-associated molecular patterns (PAMPs) from microorganisms [4]. The individual TLR-members have specialized to detect individual PAMPs from microbes, for instance, endotoxin interacts with TLR4 and microbial nucleotides, *i.e.*, double stranded RNA, single stranded RNA, double stranded DNA, with TLR3, -7 and -9, respectively. Upon recognition with their ligands, TLRs interact with up to four different adaptor molecules via cytoplasmic receptor TIR-domains, which are located at the C-terminus of TLRs and the adaptor TIR-domain containing molecules MyD88, TIR-associated protein or MyD88 Adaptor-like (TIRAP/MAL), the TIR-domain containing adaptor protein-inducing IFN-β (TRIF) and TRIF-related adaptor molecule (TRAM). Different bacterial pathogens like UPECs including the CFT073 strain; *Salmonella enterica* subsp. *enterica* Serovars Enteritidis, Dublin, Gallinarum; *Yersinia* spp.; *Brucella* spp.; *Staphylococous aureus* (*S. aureus*) MSSA476 ; and *Enterococcus faecalis* (*E. faecalis*), but also non-pathogens such as *Paracoccus denitrificans* harbor genes, which encode proteins containing a TIR-domain (Table 1) [5,6,7,8,9,10,11,12,13]. We explored earlier that TcpC is located within a *serU* t-RNA island of CFT073 [7]. Within extraintestinal pathogenic *E. coli* strains the gene is only found in the phylogenetic group B2 and is strongly associated with the presence of the main pathogenicity island [14]. Similarly, the genes encoding the *Brucella* TIR-containing protein B (TcpB) and the *Yersinia pseudotuberculosis* (*Y. pseudotuberculosis*) serotype I TcpYI protein are located within *phe* t-RNA islands [7,11]. The amino acid sequence of bacterial TIR-domains is not very similar to the eukaryotic TIR-domains, however key functional signaling domains are conserved [12]. Moreover, their tertiary structure, which will be discussed in more detail below, shows an arrangement of alternating β-sheets and α-helices, which is typical for eukaryotic TIR-domains.

### 2.2. Function of TcpC and Other Bacterial Tcps

TLRs initiate a broad defense program of the innate immune system to orchestrate the elimination of the invading pathogen. Several groups demonstrated that Tcps are able to interfere with this fundamental process of host defense. Thus, TlpA increased the bacterial load in the spleen and lethality of mice infected orally with *Salmonella enterica subspecies enteria* Serovar Enteritidis [6]. Similarly, TcpYI raised bacterial numbers in the spleen of mice intraperitoneally infected with *Y. pseudotuberculosis* serotype I [11]. Mice infected with the *tirS*-containing *S. aureus* strain MSSA476 lost more weight and demonstrated higher bacterial numbers in different organs than mice infected with the *tirS*-deficient MSSA476 strain [13]. TcpC increased the bacterial load of CFT073 in the urinary tract of infected mice and caused kidney abscesses [7] and TcpB from *Brucella melitensis* (*B. melitensis*) augmented the spread of *B. melitensis* in mice [15]. *In vitro*, TcpC and TlpA increased the intracellular amount of CFT073 and *Salmonella* Enteritidis, respectively [6,7]. The influence of Tcps on immune responses became evident by their ability to impair the release of cytokines. Thus, TcpC and TcpB impaired TNF secretion by macrophages and TcpC prevented IL-6 release by epithelial cells [7]. Moreover, it was demonstrated that TlpA, TcpC and TcpB impair the activation of the transcription factor NF-κB, which participates in the transcriptional control of many cytokine genes [6,7,16]. As the presence of the TIR-domain suggested that Tcps interfere with TLR-signaling, NF-κB reporter assays were used to analyze this possibility. The results revealed that TlpA, TcpC, TcpB and TirS interfered with TLR2 or -4 induced activation of the transcription factor [6,7,13]. Taken together, TcpC and other Tcps raised bacterial numbers *in vivo* and impaired host immune responses. Thus, the data support a role of bacterial Tcps to subvert immune responses of the host. However, based on their broad phylogenetic distribution, which also includes environmental microorganisms, microbial TIR-domains may additionally serve more general functions as protein–protein interaction domains [17].

### 2.3. Interaction of TcpC and Other Bacterial Tcps with TLR-Components

These results prompted the investigation of the TLR-Tcp interaction on the molecular level. Immuno-precipitation and pull-down assays revealed that MyD88 was the target of several Tcps. Thus, TcpC, TcpB, YpTIR and TcpF from CFT073, *B. melitensis*, *Yersinia pestis* and *E. faecalis*, respectively, bound to MyD88 but also PdTLP from the non-pathogenic *Paracoccus denitrificans* interacted with this central adaptor molecule of the TLR-signaling cascade [7,9,10,16,24]. Moreover, a subset of Tcps targeted additional members of TLRs or their adaptors. Thus, TcpC also interacted with TLR4 and TcpB with TIRAP [16,19,21,25]. Since TLR4 is an important recognition molecule during urinary tract infections [26], which activates NF-κB MyD88-dependently but also MyD88-independently via TRIF, the additional interaction of TcpC and, as shown by Alaidarous *et al.*, TcpB with TLR4 impaired the latter signaling pathway as well [16,18,19,27,28].

Eukaryotic TIR domains consist of five parallel β-strands, which are termed βA-E and are surrounded by alternating α-helices termed αA-E [27]. Loops connect alternating β-strands and α-helices and are designated according to their neighboring secondary structures. For instance, the BB-loop connects the βB-strand with the αB-helix. We previously determined the crystal structure of the MyD88 TIR-domain and demonstrated that the arrangement in space of these loops differs between the adaptor MyD88 and the receptors TLR1 and 2 [19]. To investigate the two interactions of TcpC with MyD88 and TLR4 in more detail, we generated two peptides from the BB- and DD-loop of the TcpC TIR-domain. Both peptides blocked endotoxin-induced TNF-secretion by bone marrow-derived macrophages [19]. Interestingly, we found that the BB-loop peptide interacted with TLR4, while the DD-loop peptide with MyD88, suggesting that TcpC used different surface structures of the molecule to bind to the two TLR-components [19]. We also mapped the TcpC binding site of MyD88 by nuclear magnetic resonance (NMR). Measuring the hetero-nuclear single quantum coherence spectra of the ^15^N-labeled MyD88-TIR domain in the absence or presence of TcpC, this approach revealed that the TcpC binding site of MyD88 included the amino acids M157, S244, K250, R269, F270, T272, V273, C280 and W286, which is predominantly located near CD-, DE- and EE-loops on one face of the MyD88-TIR domain [19]. The results also revealed that the C280 residue appeared to be of central importance of the TcpC-MyD88-TIR domain interaction. To confirm the relevance of C280 functionally, we generated the mutant MyD88-TIR domain C280S and found that it was still signaling competent, but TcpC could no longer impair the signaling activity of this mutated molecule [19]. These findings indicated that C280 is crucial for the binding of TcpC to MyD88 but not for the signaling ability of MyD88 as TLR-adaptor. 

### 2.4. Structure of Bacterial Tcps

**Figure 1:**
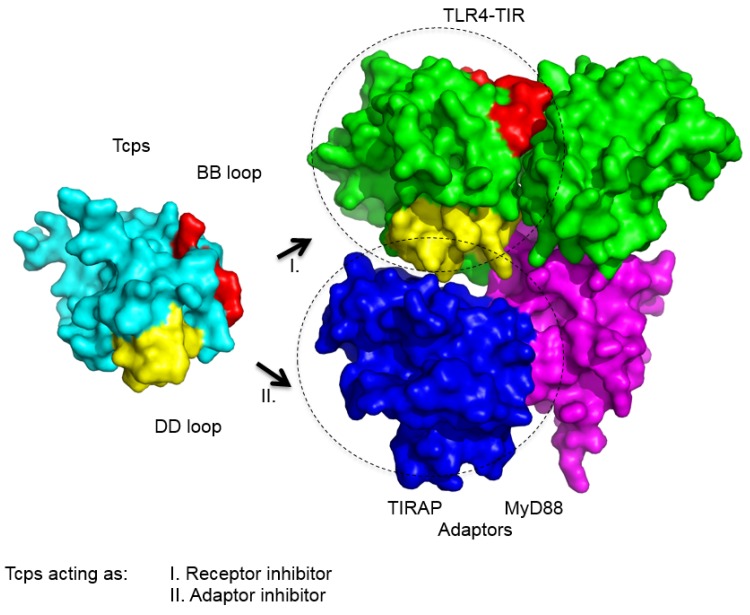
Scheme of the interactions of Tcps with TLRs and TLR-adaptors. The surface model illustrates the interaction of Tcps with the TIR-domain of TLR4 and the adaptor- molecules MyD88 or TIRAP. Thus, Tcps may block the TLR-signaling cascade as an TLR4-receptor inhibitor and/or MyD88 or TIRAP adaptor inhibitor as indicated. Color code: cyan = Tcps, green = TLR4-TIR homodimer, yellow = DD loop region, red = BB loop, blue = TIRAP, magenta = MyD88.

Our efforts to elucidate the crystal structure of TcpC have failed so far; however, crystal structures of PdTLP and TcpB were reported recently [16,20,21,24]. PdTLP was the first bacterial Tcp whose crystal structure was resolved. The analysis at 2.5-Å resolution revealed that the three-dimensional fold of its TIR-domain was identical to the ones observed for human TLRs and MyD88 [24]. Moreover, the structure shows a dimerization interface involving the DD-loop and EE-loop residues. For comparison the structure of TLR10 also revealed dimer formation of the TIR-domains where amino acid residues from the BB-loop are critical [29,30]. Our own results regarding the crystal structure of the TcpB-TIR domain show that it is composed of five alternating β-strands and α-helices similar to eukaryotic TIR-domains [21]. A structural similarity search revealed TIRAP as its closest eukaryotic neighbor [21,31,32,33] and, as described above, TcpB interacts with TIRAP. Similar to TIRAP, TcpB shares the ability to bind to phospholipids, which enables TcpB to locate to the plasma membrane of host cells [15]. The phospholipid binding motif of TcpB was mapped to the N-terminal region involving the amino acids 45-KKRADIAKK-53. In addition, TcpB also contains a microtubule-binding site enabling the molecule to associate with microtubules [15]. Moreover, our data suggest that TcpB forms a symmetric dimer, which is mediated by the DD- and EE-loop and is, thus, strikingly similar to the PdTLP-dimer. These observations were further supported by our own analysis of the interface interaction using Hydrogen Deuterium Exchange and mass spectrometry and were also reported by others [16,20]. Together, the structural results support a model of molecular mimicry of bacterial Tcps with eukaryotic TIR-domains in order to interfere with TLR-signaling (Figure 1).

### 2.5. TIR-Domains from Bacterial Tcps, TLRs and TLR-Adaptors Differ from Each Other

Although the basic arrangement of secondary structure β-strands and α-helices of bacterial and eukaryotic TIR-domains is very similar, a detailed structural comparison of the TIR-domain loop position of bacterial Tcps, TLRs and TLR adaptors revealed that these three TIR-domain containing subfamilies exhibit unique conformations of corresponding bacterial, receptor and adaptor loop positions, which may be important for function. For example, the position of the well characterized BB-loop differed substantially between the bacterial Tcps, PdTLP and TcpB, the TLR adaptor molecules, MyD88 and TIRAP, and the TLRs, TLR1, 2 and 10 [21]. Although bacterial Tcps mimic the overall fold of eukaryotic TIR-domains, they nevertheless contain unique confirmations of substructures of the TIR-domain. The functional meaning of these differences remains to be determined. More extensive structural comparisons of TIR-domains from bacteria, mammals [34] and plants [28] were recently published and are referred to for further reading. 

## 3. Conclusions

By molecular mimicry bacteria interfere with their recognition by host TLRs. A substantial part of UPECs harbor TcpC in their genomes. In case of CFT073, TcpC was shown to increase bacterial burden *in vivo* and to induce urinary tract disease like kidney abscesses in mice by impairing TLR-signaling. This principle of virulence is spread among a variety of human pathogens but also non-pathogens. TcpC is secreted by UPECs and this process can be blocked by the efflux pump inhibitor phenylalanine-arginine-β-naphtylamide (PAβN) demonstrating the principle possibility to neutralize this new virulence factor. Therefore, deeper knowledge of the mechanisms involved in TcpC secretion might offer new target structures for small molecules to block this process and by that a strategy to develop pathogen-specific treatment strategies. This strategy would also reduce the risk of the development of antibiotic resistance in the human commensal flora, since most of these bacteria lack *tcpC* genes. 

Autoimmune diseases are characterized by inflammatory processes of non-infectious cause. It is assumed that endogenous ligands such as β-defensins or self DNA/RNA from necrotic cells trigger TLRs and by that inflammation is maintained [35]. Thus, substances based on bacterial Tcps may offer new treatment possibilities to reduce inflammation characterizing these auto-inflammatory diseases.

## Figures and Tables

**Table 1:** Function and eukaryotic interaction partners of bacterial Tcps.

Bacterial TIR Protein	Organism	TIR Protein Function *in vitro*	TIR Protein Function *in vivo*	Protein Interaction with	Protein Structure	References
TcpC	*Escherichia coli* CFT073	suppresses NF-κB activation, promotes bacterial survival in RAW264.7 macrophages	promotes virulence in UTI mouse model (bacterial burden, kidney disease)	MyD88, TLR4	not known	Cirl *et al.*, 2008 [7]; Yadav *et al.*, 2010 [18]; Snyder *et al.*, 2013 [19]
TcpB (Btp1/BtpA)	*Brucella* sp.	suppresses NF-κB activation, inhibition of dendritic cell maturation	promotes virulence during early stages of infection in mice (systemic spread)	MyD88, TIRAP, TLR4, Microtubule	PDB IDs: 4C7M, 4LZP, 4LQC	Cirl *et al.*, 2008 [7]; Salcedo *et al.*, 2008 [8]; Radhakrishnan *et al.*, 2009 [15]; Kaplan-Türköz *et al.*, 2013 [20]; Snyder *et al.*, 2014 [21]; Alaidarous *et al.*, 2014 [16];
BtpB	*Brucella* sp.	suppresses TLR2, TLR4, TLR5, TLR9 mediated activation of NF-κB	promotes virulence in mice (survival)	not known	not known	Salcedo *et al.*, 2013 [22];
TlpA	*Salmonella enterica* serovar *Enteriditis*	suppresses NF-κB activation, promotes bacterial survival in human THP1 macrophages	promotes virulence in mice (bacterial survival, burden and lethality)	not known	not known	Newman *et al.*, 2006 [6];
TcpYI	*Yersinia pseudotuberculosis*	promotes bacterial survival in murine macrophages	promotes virulence in mouse model of peritonitis (survival inside the spleen)	not known	not known	Nörenberg *et al.*, 2013 [11];
YpTIR	*Yersinia pestis*	suppresses NF-κB activation	no role in virulence	MyD88	not known	Rana *et al.*, 2011 [10];
TcpF	*Enteroccocus faecalis*	suppresses NF-κB activation	promotes virulence in mice (bacterial burden)	MyD88	not known	Kraemer *et al.*, 2014 [23]; Zou *et al.*, 2014 [9];
TirS	*Staphylococcus aureus* MSSA476	suppresses NF-κB and MAP-kinase activation	Promotes virulence in mice (organ co-lonization)	not known	not known	Askarian *et al.*, 2014 [13];
PdTlp	*Paracoccus denitrificans*	not known	not known	MyD88, TLR4	PDB ID: 3H16	Low *et al.*, 2007 [5]; Chan *et al.*, 2009 [24];
